# Identification of independent risk factors for restenosis following bare-metal stent implantation: Role of bare-metal stents in the era of drug-eluting stents

**DOI:** 10.3892/etm.2013.1212

**Published:** 2013-07-10

**Authors:** CHANG-BUM PARK, HOON-KI PARK

**Affiliations:** 1 Department of Internal Medicine, Graduate School of Medicine, Kyung Hee University Hospital at Gangdong, Kyung Hee University, Seoul 134-727, Republic of Korea; 2 Department of Internal Medicine, Seoul Veterans Hospital, Seoul 134-791, Republic of Korea

**Keywords:** stent, coronary angioplasty, restenosis

## Abstract

In the era of drug-eluting stents (DESs), the ability of clinicians to predict which patients have a low risk of coronary restenosis following bare-metal stent (BMS) implantion is likely to be of benefit. The study population consisted of 2,711 patients who underwent BMS implantation in 3,770 lesions between 1995 and 2004. With clinical and 6 month follow-up angiographic data, we retrospectively sought to identify the independent risk predictors of restenosis, applied a previously proposed prediction model and assessed the characteristics of patients with a low likelihood of coronary restenosis within 6 months of BMS implantation. A 6-month follow-up coronary angiography was performed in 65.0% of the patients who had undergone the BMS implantation and the rate of restenosis was 26.6%. Using multivariate analysis, diabetes [odds ratio (OR), 1.294; 95% confidence interval (CI), 1.094–1.483; P=0.005], current smoking (OR, 1.294; 95% CI, 1.094–1.483; P=0.002), a reference vessel diameter of <3.25 mm (OR, 1.238; 95% CI, 1.021–1.501; P<0.001), a lesion length of >30 mm (OR, 1.645; 95% CI, 1.336–2.026; P<0.001), ostial lesion (OR, 1.858; 95% CI, 1.437–2.402; P<0.001), post-stenting minimal luminal diameter (OR, 0.576; 95% CI, 0.484–0.685; P<0.001) and bifurcation lesion (OR, 1.353; 95% CI, 1.070–1.711; P=0.012) were identified as significant independent predictors of restenosis. However, the accuracy of the prediction obtained with the current model, which used the clinical and angiographic variables correlated with the risk of restenosis, was poor. Various clinical and angiographic independent risk variables were revealed to be correlated with the risk of restenosis following BMS implantation in the present large dataset. Certain groups of patients with a relatively low risk of restenosis may be considered for BMS implantation as an alternative to DESs. However, the prediction models used at present are incomplete and further studies are required.

## Introduction

Percutaneous coronary intervention (PCI) with stent implantation has replaced balloon angioplasty, due to a reduction in the incidence of restenosis. However, an in-stent restenosis rate of 10–40% has been a significant problem in bare-metal stent (BMS) implantation (1–3). Recently-developed drug-eluting stents (DESs) have reduced the rate of restenosis and the use of DESs has therefore expanded exponentially in a wide variety of clinical and anatomical situations. However, the higher cost of DESs and the risk of stent thrombosis are major limiting factors. Therefore, a knowledge of the risk factors for restenosis in BMS implantation may be beneficial in guiding strategies such as the selection of optimal candidates for BMS and DES implantation. A study by Singh *et al*(4) suggested prediction models for the risk of restenosis, using numerous clinical, angiographic and procedural variables to rapidly assess and evaluate the apparent risk for the patient.

Therefore, with clinical and 6-month follow-up angiographic data, we retrospectively sought to identify the independent risk variables for restenosis, to evaluate the predictive ability of the model suggested by Singh *et al*(4) following BMS implantation and to identify the characteristics of patients with a low likelihood of restenosis.

## Material and methods

### Patient population and data collection

The study population consisted of patients who had undergone BMS implatation between September 1995 and August 2004. A follow-up coronary angiography was performed 6 months subsequent to the PCI with BMS implantation at tertiary teaching hospitals. Patients excluded from the angiographic follow-up were those who experienced major adverse cardiac events during the first 30 days following the procedure, including mortality, myocardial infarction and repeated revascularization, and patients who were shown to have an unsuccessful technical result (diameter stenosis ≥50%). In such cases, subsequent emergency bypass graft surgery was performed, prior to hospital discharge.

Patient data regarding age, gender, diabetes, hypertension, current smoking status, clinical diagnosis at presentation, elevated total cholesterol (≥200 mg/dl), prior myocardial infarction, prior PCI, prior bypass graft surgery and renal insufficiency (creatinine ≥2 mg/dl) were retrospectively obtained using medical records. This study was approved by the Ethics committee of Kyung Hee University Hospital at Gangdong (No: KHNMC MD IRB 2013-007).

### Angioplasty procedure

The following BMSs were used: BX Sonic or BX Velocity (Cordis, Johnson & Johnson, Warren, NJ, USA) in 30.2% of cases, NIR (Scimed Life Systems, Inc, Maple Grove, MN, USA) in 21.3% of cases, S7 and GFX (Arterial Vascular Engineering, Inc., Santa Rosa, CA, USA) in 19.0% of cases and other stents in 29.5%. Stent implantation was performed according to standard techniques and the stents were selected by the surgeon. All patients were pretreated with aspirin (100 or 200 mg/day), and ticlopidine (500 mg/day) or clopidogrel (75 mg/day) for at least one month.

A follow-up coronary angiography was performed between six and nine months following the BMS implantation. Angiographic analysis was blindly performed by two experienced angiographers who were unaware of the aim of the study. Percent diameter stenosis, minimal lumen diameter (MLD) and reference vessel size were measured prior to and following the stenting procedure, and at the follow-up, using an on-line quantitative angiographic analysis system (Philips Medical Systems, Amsterdam, The Netherlands). Angiographic measurements were made during diastole following intracoronary nitroglycerin administration, using the guiding catheter to calibrate magnification. The lesion type was classified according to the modified American College of Cardiology/American Heart Association (ACC/AHA) grading system (5). Angiographic restenosis was defined according to the binary distinction of >50% stenosis at the time of the angiographic follow-up.

### Definitions

All demographic, clinical, angiographic and procedural characteristics were retrospectively reviewed using the database. Restenosis was defined as a diameter stenosis of ≥50% occurring in the segment inside the stent or in the 5 mm segment proximal or distal to the stent at the follow-up angiography.

### Restenosis risk model

The restenosis risk model developed by Singh *et al*(4) is generated from eight clinical and preprocedural angiographic characteristics that group patients undergoing coronary intervention into four categories according to their risk of restenosis within 6 months of the procedure. The variables used have frequently been demonstrated to be strong predictors of restenosis risk and were selected on the basis of a review of previous studies. The categorization was performed as follows: A lesion length ≥20 mm was assigned four points; a C-type lesion according to the ACC/AHA classification was assigned three points; a previous history of PCI, diabetes and being a nonsmoker were each assigned two points and the presence of unstable angina and the female gender were each assigned one point. The points assigned for vessel size varied, with one point assigned for 3.5–4 mm, two points for 3–3.5 mm and three points for ≤3 mm. Patients with a score of between 0 and 2 were identified as having a 28% (95% CI, 16–40%) risk of restenosis, while patients with scores of 3–7 were predicted to have a 40% risk (95% CI, 37–43%) and those with scores of 8–14 were predicted to have a 63% risk (95% CI, 58–68%). A score of ≥15 was correlated with a 91% (95% CI, 74–100%) risk of restenosis.

The success of this model in identifying patients with restenosis has been shown to be comparable with that of other models. The area under the receiver-operator characteristics (ROC) curve was 0.63, indicating a modest discriminatory ability of the model.

### Statistical analysis

Data are expressed as the mean ± standard deviation for continuous variables, and frequencies for the categorical variables. Continuous variables were compared using an independent-samples t-test, while categorical variables were compared using a χ^2^ test. The first approach used the data to compare the predictive ability of the risk-score approach, as described previously. Initially, the baseline characteristics were screened as candidate predictor variables of restenosis. Multivariate analyses were performed to identify independent predictors of adverse events at univariate analysis (P≤0.1), and the predictors were subsequently tested to identify their multivariate predictive value [tested variables: diabetes, smoking, lesion length >30 mm, reference diameter <3.25 mm, post-stenting MLD, ostial lesion, bifurcation lesion, diffuse lesion (≥20 mm) and age ≥60 years]. No attempt was made to construct a simple score using this model. The area under the ROC curve was calculated on the basis of the estimated risks from the final model. P<0.05 was considered to indicate a statistically significant difference. In the second approach, the previously mentioned restenosis risk model was applied to patients who were positive for all of the eight variables in the procedural database, in order to estimate their risk of restenosis. The significant risk factors were identified by the logistic regression program (SPSS statistical software version 14.0; SPSS, Inc., Chicago, IL, USA). The area under the ROC curve is presented for the risk score.

## Results

### Baseline characteristics

A 6-month follow-up coronary angiography was performed in 65.0% of the patients who underwent a BMS implantation. Out of the 4,986 lesions (3,448 patients) that were treated with BMS implantation between September 1995 and August 2004, 1,216 lesions (737 patients) were excluded due to the absence of one or more of the values required to compute the score. Thus, a total of 3,770 lesions (2,711 patients) were available for analysis.

### Univariate and multivariate analysis

Restenosis occurred in 1,003 lesions (26.6%). Tables I and II show the baseline clinical characteristics in the restenosis and non-restenosis groups and Table III shows the baseline angiographic characteristics per lesion. The restenosis group was observed to have a higher incidence of diabetes than the non-restenosis group (25 versus 20%, respectively; P<0.001) and the restenosis group was more likely to smoke (46 versus 42%; P=0.013). However, the incidences of hypertension, previous PCI, current unstable angina and acute myocardial infarction did not differ significantly between the two groups. With regard to the angiographic characteristics in the restenosis and non-restenosis groups, lesion length >30 mm (13 versus 6%, respectively; P<0.001), reference diameter ≤3.25 mm (65 versus 49%, respectively; P<0.001), post-stenting MLD (3.05±0.55 versus 3.27±0.59 mm, respectively; P=0.026), ostial lesion (11 versus 8%, respectively; P=0.013), diffuse lesion (15 versus 9%, respectively; P<0.001) and bifurcated lesion (12 versus 9%, respectively; P=0.009) were all correlated with a higher incidence of restenosis.

Using multivariate analysis, diabetes (OR, 1.294; 95% CI, 1.083–1.547; P=0.005), smoking (OR, 1.274; 95% CI, 1.094–1.483; P=0.002), reference vessel diameter ≤3.25 mm (OR, 1.238; 95% CI, 1.021–1.501; P<0.001), lesion length >30 mm (OR, 1.645; 95% CI, 1.336–2.026; P<0.001), ostial lesion (OR, 1.858; 95% CI, 1.437–2.402; P<0.001), post-stenting MLD (OR, 0.576; 95% CI, 0.484–0.685; P<0.001) and bifurcated lesion (OR, 1.353; 95% CI, 1.070–1.711; P=0.012) were revealed to be significant independent predictors of restenosis, as shown in Table IV.

### Procedures by risk score

Using the risk prediction model suggested by Singh *et al*(4) in the Prevention of Restenosis With Tranilast and Its Outcomes (PRESTO) trial, which is shown in Table V, four subgroups were constructed with risk scores of 0–2, 3–7, 8–14 and ≥15. A risk score of 0–2 (n=335) was correlated with a 18.5% risk of restenosis in the present study, compared with 28% in the PRESTO trial, while a score of 3–7 (n=2,065) was correlated with a 24.4% restenosis risk in the present study, compared with 40% in the PRESTO trial. Scores of 8–14 (n=1,337) were correlated with 31.3 and 63% risks in the present study and the PRESTO trial, respectively, while a risk score of ≥15 (n=33) was correlated with 60.6 and 91% risks in the present study and the PRESTO trial, respectively (Table VI shows present study risk data). The area under the ROC curve was 0.565, which indicates that the model has a poor discriminatory ability.

### Identification of groups at low risk of restenosis

The observed 6-month restenosis rates according to lesion length and reference diameter are presented in Table VII, while the rates in a low-risk group of patients, according to independent risk variables and risk score, are summarized in Table VIII. A total of eight subgroups had an observed 6-month restenosis rate of <20%; however, none of the groups had an observed restenosis rate of <10%.

## Discussion

This study reflected the interventions of a large number of surgeons in the treatment a wide variety and relatively high-volume of patients. The study showed that a variety of clinical and angiographic variables were correlated with restenosis subsequent to BMS implantation, which was consistent with the results from previous studies (6–12). A total of eight subgroups of patients were identified as being at a low risk of restenosis, with restenosis rates of <20%; by contrast, there were no subgroups with an observed 6-month restenosis rate of <10%. However, the current risk model, which solely used clinical and angiographic variables, demonstrated a poor ability to predetermine the risk of angiographic restenosis in actual clinical practice.

Coronary stenting has become a standard therapy for coronary artery disease, due to the simplicity of the procedure and its favorable long-term outcomes. However, restenosis affects a significant number of patients who undergo BMS implantation. Over the past few years, DESs have been shown to markedly reduce the occurrence of restenosis in selected patients. More than 80% of percutaneous interventions in the United States in 2004 were performed with the use of DESs coated with sirolimus or paclitaxel (13).

The present study showed that the angiographic restenosis rate was 26.6% and identified a number of clinical and angiographic predictors, such as smoking, diabetes, ostial lesion, bifurcation lesion, reference diameter ≤3.25 mm, lesion length >30 mm and post-procedural MLD. A number of previously reported studies (6–12) have described similar angiographic restenosis rates, ranging from 19 to 29.2% at 6 months following the stent implantation, in addition to similar baseline and procedural characteristics correlating with the risk of subsequent restenosis following the implantation of a BMS.

Singh *et al*(4) evaluated 1,312 patients, each with a single lesion which had been treated successfully by PCI and who were enrolled in the angiographic substudy of PRESTO. The PRESTO database aimed to classify the risk of restenosis, and utilized clinical and angiographic variables that had been identified in studies prior to the procedure. The authors also used a bootstrap approach to evaluate a model derived solely from the PRESTO database. However, when this risk-score model was applied to the present large database, certain problems were encountered. This included the fact that although the selected factors demonstrated significant correlations with the development of subsequent restenosis, there was only a modest ability to discriminate between patients with or without restenosis. Furthermore, the inclusion of being a nonsmoker as one of the risk-score variables was questionable, since in the present study smoking was evaluated to be one of independent risk variables (14). Moreover, smoking has been demonstrated to be associated with adverse effects on long-term survival following PCI in previous studies (14,16), and the ‘Smoker's paradox’ has been shown to be associated with other confounding factors, such as age and socioeconomic status.

There have been numerous studies (6–12) investigating the predictive risk variables for restenosis; however, there have been relatively few studies concerning the risk stratification of restenosis. Kettlekamp *et al*(17) used the Mid America Heart Institute (MAHI) restenosis risk model (18) to predict restenosis. The authors used clinical variables and the frequency of angina in the prediction, although they did not use angiographic variables, and compared the current referral patterns of patients requiring coronary revascularization to PCI or bypass graft as a function of the underlying risk for restenosis. However, the predictive accuracy of the MAHI restenosis risk model was not described. Kastrati *et al*(8) revealed the strongest multivariate predictors for in-stent restenosis to be diabetes, multiple stents and a post-stent MLD of <3 mm. The classification and regression tree (CART) model was used, and the score was then constructed by a simple arithmetic sum of the number of variables present. This study had an incomplete angiographic follow-up and did not describe the predictive accuracy of the model. Ellis *et al*(19) studied 5,239 patients identified with a low-risk of 9-month revascularization and created models using logistic regression and Cox multiple hazard regression analyses. The predictive discrimination of the model was modest (area under the ROC curve=0.65). This study was retrospective and did not distinguish the target lesion from non-target vessel revascularization. Therefore, we hypothesized that the current prediction models for restenosis involving angiographic and demographic predictors were inadequate and required further investigation.

The present study did not identify any groups of patients who were likely to have less than a 10% risk of restenosis within 6 months of the index procedure. However, eight groups were observed to have a risk of less than 20%, and, therefore, multiple relatively low-risk population groups were identified. The result was consistent with that from a previous report, although the low-risk subgroups were different (19).

This study demonstrated several limitations, with the principle limitation being that the study was a retrospective, non-randomized, single center analysis. Another limitation was that the use of BMSs has decreased since 2003, following the introduction of DESs, and therefore there may have been a selection bias with regard to the use of BMSs.

In conclusion, a variety of clinical and angiographic independent risk variables were shown to be correlated with a risk of restenosis following BMS implantation in a large dataset. The prediction accuracy of the model using the clinical and angiographic variables correlated with the risk of restenosis was poor.

## Figures and Tables

**Table I:** Baseline clinical characteristics per patient.

	Restenosis		
		
Characteristics	Yes (n=849)	No (n=1862)	P-value
Age (years)	58.1±9.6	57.2±9.6	0.016
Male, n (%)	632 (74.4)	1332 (71.5)	0.415
Medical history, n (%)			
Diabetes	206 (24.3)	328 (17.6)	<0.001
Hypertension	325 (38.3)	732 (39.3)	0.373
Smoking	390 (45.9)	781 (41.9)	0.125
Hypercholesterolemia (≥200 mg/dl)	314 (37.0)	725 (38.9)	0.179
Previous MI	48 (5.7)	137 (7.4)	0.079
Previous PCI(s)	26 (3.1)	22 (1.2)	0.001
Previous CABG	4 (0.5)	4 (0.2)	0.273
Unstable angina pectoris, n (%)	415 (48.9)	889 (47.7)	0.925
Acute myocardial infarction, n (%)	172 (20.3)	407 (21.9)	0.235
Ejection fraction percent	59.6±9.1	60.1±9.3	0.305
Renal insufficiency (creatinine ≥2.0 mg/dl), n (%)	8 (0.9)	20 (1.1)	0.717
Use of glycoprotein IIb/IIIa inhibitor, n (%)	37 (4.4)	110 (6.0)	0.078

Age and ejection fraction percent data are mean ± standard deviation. MI, myocardial infarction; PCI, percutaneous coronary intervention; CABG, coronary artery bypass graft.

**Table II:** Baseline clinical characteristics per lesion.

	Restenosis		
		
Characteristics	Yes (n=1003)	No (n=2767)	P-value
Age (years)	58.3±9.6	57.6±9.5	0.673
Male, n (%)	750 (74.7)	2040 (73.7)	0.516
Medical history, n (%)			
Diabetes	254 (25.3)	542 (19.6)	<0.001
Hypertension	385 (38.4)	1134 (41.0)	0.151
Smoking	461 (46.0)	1147 (41.5)	0.013
Hypercholesterolemia (≥200 mg/dl)	379 (37.8)	1118 (40.4)	0.147
Previous MI	57 (5.7)	189 (6.8)	0.207
Previous PCI(s)	30 (3.0)	61 (2.2)	0.164
Previous CABG	5 (0.5)	7 (0.3)	0.322
Unstable angina pectoris, n (%)	484 (48.3)	1346 (48.6)	0.833
Acute myocardial infarction, n (%)	196 (19.5)	584 (21.1)	0.295
Ejection fraction percent	59.6±9.3	59.8±9.9	0.433
Renal insufficiency (creatinine ≥2.0 mg/dl), n (%)	8 (0.8)	31 (1.1)	0.387
Use of glycoprotein IIb/IIIa inhibitor, n (%)	43 (4.3)	147 (5.3)	0.203

Age and ejection fraction percent data are mean ± standard deviation. MI, myocardial infarction; PCI, percutaneous coronary intervention; CABG, coronary artery bypass graft.

**Table III:** Baseline angiographic characteristics per lesion.

	Restenosis		
		
Characteristics	Yes (n=1003)	No (n=2767)	P-value
Lesion length, n (%)			
≤15 mm	276 (27.5)	922 (33.3)	0.001
15.1–30 mm	600 (59.8)	1677 (60.6)	0.663
>30 mm	127 (12.7)	168 (6.1)	<0.001
Pre-procedural MLD (mm)	0.79±0.59	0.85±0.75	0.579
Post-procedural MLD (mm)	3.05±0.55	3.27±0.59	0.026
Reference diameter, n (%)			
≤2.75 mm	268 (26.7)	462 (16.7)	<0.001
2.76–3.25 mm	380 (37.9)	896 (32.4)	0.002
>3.25 mm	355 (35.4)	1409 (50.9)	<0.001
Lesion characteristics, n (%)			
Ostial lesion	108 (10.8)	226 (8.2)	0.013
Bifurcated lesion	124 (12.4)	261 (9.4)	0.009
Diffuse lesion	147 (14.7)	262 (9.5)	<0.001
Chronic total occlusion	18 (1.8)	31 (1.1)	0.106
Calcified lesion	31 (3.1)	67 (2.4)	0.254
*De novo* lesion	963 (96.0)	2658 (96.1)	0.674
ACC/AHA lesion type, n (%)			0.808
Type A, B1, B2	658 (65.6)	1827 (66.0)	
Type C	345 (34.4)	940 (34.0)	
Vessel			
Location, n (%)			0.889
Left anterior descending	492 (49.1)	1344 (48.6)	
Left circumflex	172 (17.1)	473 (17.1)	
Right coronary	286 (28.5)	791 (28.6)	
No. of involved arteries, n (%)			0.610
Single	485 (48.4)	1312 (47.4)	
Multivessel disease	518 (51.6)	1455 (52.6)	

Pre- and post-procedural MLD data are mean ± standard deviation. MLD, Minimal luminal diameter; ACC/AHA, American College of Cardiology/American Heart Association.

**Table IV:** Independent risk variables for restenosis, identified using multivariate analysis.

Variables	OR	95% CIs	P-value
Smoking	1.274	1.094–1.483	0.002
Diabetes	1.294	1.083–1.547	0.005
Ostial lesion	1.858	1.437–2.402	<0.001
Bifurcation	1.353	1.070–1.711	0.012
Post-procedural MLD	0.576	0.484–0.685	<0.001
Reference diameter (≤3.25 mm)	1.238	1.021–1.501	<0.001
Lesion length (>30 mm)	1.645	1.336–2.026	<0.001

OR, odds ratio; CI, confidence interval; MLD, Minimal luminal diameter.

**Table V:** Risk-score model derived from variables selected from previously published studies.

Variable	Odds ratio (95% CIs)	P-value	Integer score
Lesion length >20 mm	2.07 (1.34, 3.21)	0.001	4
ACC/AHA type C lesion	1.81 (1.26, 2.59)	0.001	3
Previous PCI	1.46 (1.14, 1.88)	0.003	2
Treated diabetes	1.41 (1.03, 1.92)	0.034	2
Nonsmoker	1.39 (1.05, 1.83)	0.022	2
Vessel size			
>4 mm	1.00 (reference)		
3.5–4 mm	1.18 (0.55, 2.53)	0.680	1
3–3.5 mm	1.44 (0.71, 2.94)	0.317	2
≤3 mm	1.76 (0.87, 3.54)	0.115	3
Unstable angina	1.19 (0.94, 1.51)	0.147	1
Female gender	1.15 (0.87, 1.52)	0.317	1

CIs, Confidence intervals; ACC/AHA, American College of Cardiology/American Heart Association; PCI, percutaneous coronary intervention.

**Table VI:** Summary table for observed and predicted risk by risk score.

Risk score range	Applied no. of patients	Observed risk of restenosis (%)
0–2	335	18.5
3–7	2060	24.4
8–14	1335	31.3
15+	33	60.6

**Table VII:** Observed 6-month restenosis rates (%) according to lesion length and reference diameter.

Reference diameter (mm)	Lesion length (mm)		

	<10	10–30	>30
<2.75	14/36 (38.9)	226/644 (35.1)	28/50 (56.0)
2.75–3.25	18/41 (43.9)	299/804 (27.1)	63/132 (47.7)
>3.25	28/152 (18.4)	291/1499 (19.4)	36/113 (31.9)

**Table VIII:** Observed 6-month restenosis rates in low-risk subgroups of patients using multivariate independent risk factors and the risk-score model.

Variables	Observed restenosis rate
Multivariate independent factors (%)	
No risk factor	83/544 (15.3)
Diabetes only	22/138 (15.9)
Smoking only	94/503 (18.7)
Bifurcation only	11/47 (19.0)
Reference vessel diameter >3.25 mm and lesion length ≤30 mm	319/1651 (19.3)
Sum of risk scores (%)	
0	6/32 (18.8)
1	19/99 (19.2)
2	37/204 (18.1)
